# Integrating Interactive Computational Modeling in Biology Curricula

**DOI:** 10.1371/journal.pcbi.1004131

**Published:** 2015-03-19

**Authors:** Tomáš Helikar, Christine E. Cutucache, Lauren M. Dahlquist, Tyler A. Herek, Joshua J. Larson, Jim A. Rogers

**Affiliations:** 1 Department of Biochemistry, University of Nebraska–Lincoln, Lincoln, Nebraska, United States of America; 2 Department of Biology, University of Nebraska–Omaha, Omaha, Nebraska, United States of America; 3 Department of Mathematics, University of Nebraska–Omaha, Omaha, Nebraska, United States of America; University of British Columbia, CANADA

## Abstract

While the use of computer tools to simulate complex processes such as computer circuits is normal practice in fields like engineering, the majority of life sciences/biological sciences courses continue to rely on the traditional textbook and memorization approach. To address this issue, we explored the use of the Cell Collective platform as a novel, interactive, and evolving pedagogical tool to foster student engagement, creativity, and higher-level thinking. Cell Collective is a Web-based platform used to create and simulate dynamical models of various biological processes. Students can create models of cells, diseases, or pathways themselves or explore existing models. This technology was implemented in both undergraduate and graduate courses as a pilot study to determine the feasibility of such software at the university level. First, a new (In Silico Biology) class was developed to enable students to learn biology by “building and breaking it” via computer models and their simulations. This class and technology also provide a non-intimidating way to incorporate mathematical and computational concepts into a class with students who have a limited mathematical background. Second, we used the technology to mediate the use of simulations and modeling modules as a learning tool for traditional biological concepts, such as T cell differentiation or cell cycle regulation, in existing biology courses. Results of this pilot application suggest that there is promise in the use of computational modeling and software tools such as Cell Collective to provide new teaching methods in biology and contribute to the implementation of the “Vision and Change” call to action in undergraduate biology education by providing a hands-on approach to biology.

## Introduction

The enormous complexity that recent research has revealed in biological and biochemical systems has resulted in the emergence of mathematical modeling and computer simulations as an integral part of biomedical research. This provides researchers with new tools to understand the role of emergent properties in healthy and diseased cells, to generate new hypotheses, and even screen potential pharmaceuticals for cross-reactivity and potential targets [1–3].

Given the fact that the field is undergoing a shift in the basic way the functions of these dynamical systems/networks are understood, it is essential for biology education to evolve in order to reflect these changes [4,5]. It is vital for students to learn about these structures and the resultant emergent properties that are not obvious from looking at static pictures in textbooks. Furthermore, the National Science Foundation and the American Association for the Advancement of Science have initiated a call to action, “Vision and Change” [6], that aims to transform undergraduate biology education by incorporating computational methods and by introducing key core competencies including simulation and modeling. A number of efforts have already been initiated in this direction, including problem-based learning in the undergraduate setting [7], translational approaches (i.e., having students serve as researchers in the classrooms to investigate biological problems and identify solutions), as well as those led by Carl Wieman of the Carl Wieman Institute [8] and other leaders in foundational learning (e.g., [9,10]).

Our group has also attempted to address this issue using our recently developed and released modeling platform called Cell Collective [11,12]. The platform enables scientists to create, simulate, and analyze large-scale computational models of various biological systems without the need to enter/modify any mathematical expressions and/or computer code. Because accessibility to modeling for a wide audience is the key ingredient of the technology, the platform lends itself to application in a classroom setting. Specifically, students can create, simulate, and analyze then break and re-create and re-analyze dynamical models to understand major biological processes. The collaborative nature of the Web-based environment enables students to easily collaborate inside and outside of the classroom in a meaningful way. The types of biological processes that can be explored with Cell Collective are virtually unlimited; students can model biological processes including, but not limited to, cellular development, cellular differentiation, cell-to-cell interactions, disease pathogenesis, the effects of various treatments on disease, etc.

Herein, we discuss two different applications of the Cell Collective’s interactive technology as a tool to facilitate hands-on, creative learning in the classroom and allow students to apply their knowledge in real-time. The first is using Cell Collective in a dedicated course (In Silico Biology) designed around the use of the technology, and the second involves introducing the technology as a supplement to existing, traditional biology courses. Both applications have been subjected to initial testing in a variety of undergraduate settings, and the results indicate that both methods were successful in increasing both understanding of and enthusiasm for complex biological systems in undergraduate student populations.

## A New Course Designed for Integrated Learning of Biological and Computational Concepts

In Silico Biology is a course that was designed de novo to use Cell Collective as the central tool for teaching students complex biochemical systems by recreating them in silico. The individual objectives of the course include helping students to expand their analytical skills and become interested in computational sciences, learn to actively read primary journal articles, critically analyze and interpret data, and use interactive computational models to learn about biological networks. This is facilitated throughout the course via three major topics and strategies incorporated into the course:

### 1. Introduction of biological concepts from a systems perspective

The focus of the biological component of the In Silico Biology course is on complex networks found in biological systems. A series of lectures at the beginning of the semester provide students with the foundation of molecular biology of the cell, including the principles of intra- and intercellular signal transduction. During this session, students also learn to think about biochemical protein regulatory mechanisms from a holistic perspective; that is, rather than focusing on individual protein–protein interactions, students are expected to research and understand the overall regulatory mechanism of a given protein while taking into account most known interaction partners.

**Fig 1:**
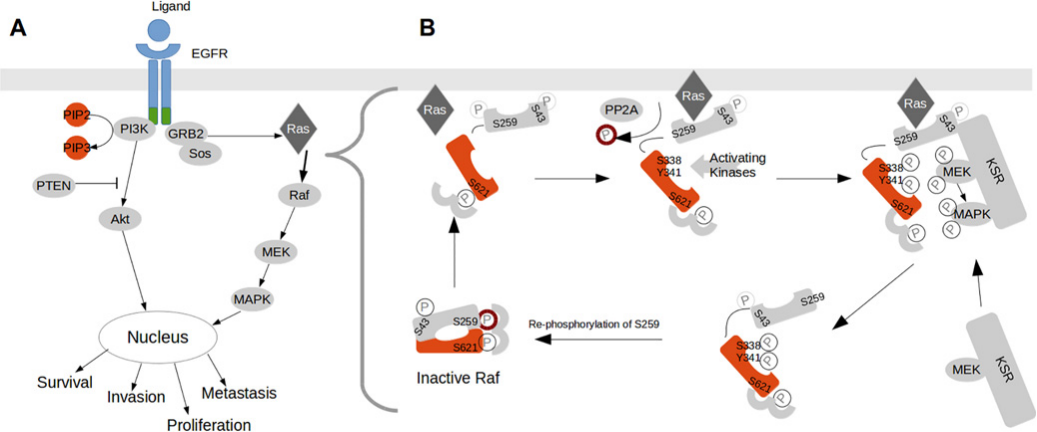
Comparison of linear and systems representation of Raf regulation. A) Traditional (linear) representation of MAPK signaling [13]. B) Detailed regulatory mechanism of Raf regulation that takes into account the role of most Raf interaction partners.

For example, students are required to go beyond the traditional representation of the regulation of the Raf protein (Fig. 1). Raf is a key component of the mitogen activated protein kinase (MAPK) pathway, which regulates numerous cellular functions (e.g., growth, apoptosis, etc.). Students learn that Ras is only one of many components required to successfully activate, as well as deactivate, Raf via a combination of biochemical events (Fig. 1B) [13,14]. In the final part of this session, students are expected to research (from published literature) and describe the complete regulatory mechanism of an enzyme of their choice, as a system of multiple interaction components. In this session, students are also introduced to Cell Collective, and are expected to create and simulate a simple pathway model such as the one illustrated in Fig. 1A, as well as to model the regulatory dynamics of the researched enzyme. Importantly, with this approach, the students learn how to read and critically analyze primary journal articles.

(Fig. 1 adapted from [14])

### 2. Introduction to the dynamics of biological systems via computational modeling

In this part of the course, students learn the principles of the technology and modeling framework on which Cell Collective is based (Section 1 in S1 Text) [15–20]. This includes the different types of representation of Boolean functions, as well as concepts of state transition graphs, feedback loops, attractors, attractor stability, etc. All of these concepts are tied to and demonstrated in the Cell Collective platform and applied to biological examples. Students also learn about nonlinear dynamics such as bistability and oscillations associated with positive and negative feedback loops, respectively. By the end of this session, students are able to represent complex biological regulatory mechanisms as Boolean functions and create and simulate the dynamics of their corresponding models (by hand, as well as in Cell Collective). An example of a biological system well-suited to the approach is bacterial chemotaxis (Section 2 in S1 Text).

### 3. Blurring the line between education and research: incorporating meaningful undergraduate research experiences into the classroom

A large part of the course is devoted to a hands-on project during which students learn about a biological system by integrating the biological and computational concepts they learned in the course. Specifically, students select a biological network process of interest that they research, construct a computational model representation of, and study the dynamics of the process by simulating the model in Cell Collective.

From our experience, it is the learning-by-building approach that enables students to learn and appreciate the diversity and complexities of biological systems. This method fosters curiosity from the students, which keeps them motivated and active in the project. Reading the literature with an objective to create a functional computer model forces the student to truly parse and analyze the information contained in published papers in order to distill the underlying logic of the system. Section 3 in S1 Text provides an example of how students learn by reading the literature and performing virtual research.

## Incorporating Computational Modeling in Existing Life Sciences Courses

As it is not always possible or practical to create a complete course de novo, the Cell Collective platform has also been used to aid in various existing undergraduate and graduate courses. These include undergraduate/graduate (online) cancer biology, undergraduate microbiology, and graduate (online) immunology courses.

In order to facilitate the introduction of modeling into existing courses, a series of modeling modules were created; these modules are currently available in a new problem-based workbook [21] focused on cancer biology. Students utilized the models that comprise the various modules to simulate and analyze the dynamics of the biological processes as a way to visualize and reinforce the content discussed during regular lectures. The interactive nature of the technology enables students to alter any component or pathway of the process and, via instant feedback, observe the effects of the change made to the system.

The modules are complementary to the traditional method of teaching as an interactive, dynamic process with learning objectives that match the covered topic. For example, from the exercises used in the cancer biology course, learning objectives focused on 1) determining the dynamic, complex signaling processes that regulate tumor development and tumor regression and 2) the ability to illustrate feedback loops that contribute to tumor progression and regression after use of the Cell Collective. Learning objectives of the computational module used in the microbiology course centered on the life cycle of *Plasmodium* spp., which leads to the development of malaria. Specifically, after dynamically modeling and manipulating developmental processes of the *Plasmodium* lifecycle, students should be able to 1) draw and describe the complex life cycle and 2) define “vector,” “reservoir,” and “transmission.”

Table 1 provides a list of modeling modules developed so far. As an example, one of these modeling modules used for more effective learning is discussed next.

**Table 1:** List of developed modeling modules.

Example Biological Concept Taught	Course Type/Topic
Malaria lifecycle	Microbiology
Positive feedback loops	Cancer biology
Negative feedback loops	Cancer biology
Cell cycle regulation	Cancer biology
DNA damage	Cancer biology
CD4+ T cell differentiation	Cancer biology, immunology
Cell communication networks	Cancer biology

### T cell differentiation and response to pathogen

**Fig 2:**
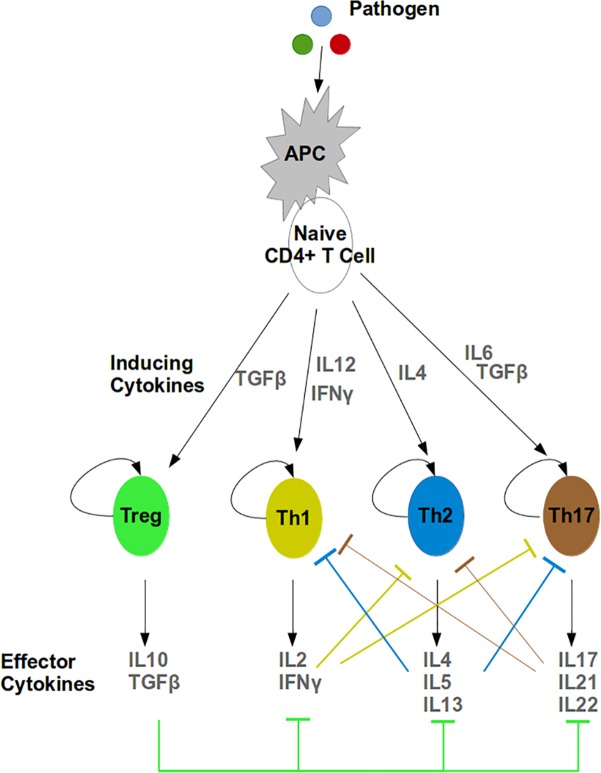
CD4+ T Cell differentiation as modeled for classroom use.

T cell differentiation is an important concept taught in many immunology courses. Precise regulation of the differentiation process of naive CD4+ T cells (a subset of T-lymphocytes) to one of the helper T cells or regulatory T cells (Tregs) is critical for the proper functioning of the immune system. At the intracellular level, the differentiation process is regulated via a wide variety of types of signaling receptors and pathways that are mutually cross-linked and form highly interconnected biochemical networks. Additionally, cytokines produced by each cell further modulate the activation and behavior of neighboring cells, as well as the entire immune system [22,23]. Hence, the complex network structures and nonlinear dynamics governing this process, via both intra- and intercellular paths, make T cell differentiation a great candidate for an interactive modeling approach. As such, a modeling module that mimics concepts and relationships (Fig. 2) was created and used to aid the learning of T cell differentiation in Cell Collective.

**Fig 3:**
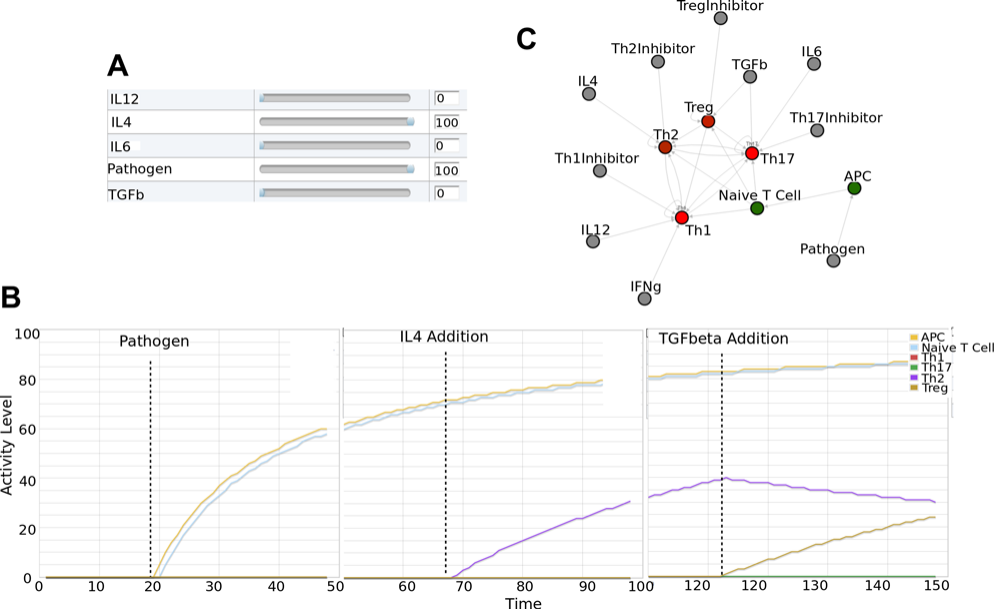
Interactive simulation of a T cell differentiation model. A) Simple sliders can be used to change the activity levels of various stimuli. B) Example of an interactive, real-time simulation. Left: Activation of Pathogen results in the stimulation of Antigen Presenting Cells and Naive T Cells. Middle: Stimulation with IL4 results in the activation of Th2 cells. Right: Addition of TGF beta stimulates Tregs, resulting in the suppression of Th2 cells. C) Network view of the changing dynamics during a real-time simulation. (Color range from bright red [inactive components] to bright green, which denotes full activity.)

An advantage to the availability of a tool such as Cell Collective is that students can alter external and internal conditions of the cell and observe real-time “output” or consequences at the molecular and/or cellular level. For example, students are asked to simulate the model by first activating antigen presenting cells (APCs) and naive T cells by introducing a “pathogen.” Pathogens can be introduced by changing a simple activity slider on the user interface (Fig. 3A). As illustrated in Fig. 3B (left), the dynamical response to the change of the environment is immediate. Students can subsequently simulate Th2 differentiation by introducing IL4 (Fig. 3B middle), as well as the effects of regulatory T cells (Treg) by activating TGF beta (Fig. 3B right). In addition to the time-series, real-time simulation output, students can view the dynamics of the entire model in a network representation in which each component of the model interactively assumes different colors based on the activity level of the component (Fig. 3C).

Students are assigned a number of similar exercises to better understand the dynamics governing T cell regulation during the activation of the immune system, including positive feedback loops and associated bistable behaviors. Note that this model is one of many possible computational model representations of T cell differentiation. Other logical models that include greater detail as to specific molecular interactions have been previously published by others [24–26], and some of these are also available in Cell Collective for simulations.

## Outcomes and Discussion

A number of efforts to incorporate computation into life science courses have been established. For example, BioQuest consortium (http://www.bioquest.org) provides access to software tools, datasets, and other materials developed by educators and developers engaged in education and research in science. Another example includes NetLogo, a programming environment for agent-based modeling that has been used to study dynamics of complex systems, as well as for teaching of complex systems in many settings (middle schools, high schools, and universities) [27]. Our approach provides a novel take on the implementation of problem-based methods in life sciences in that it offers a Web-based, systems- and network-focused, interactive, and real-time simulation-driven environment without the need for computer programming or manipulation of complex mathematical equations.

We have used a Cell Collective “learning by modeling” approach both as a stand-alone class and as a supplement to complement existing classes. In both cases, student outcomes were highly positive (Section 5 in S1 Text). Future studies will include a comprehensive study using both quasi-experimental and randomized control groups to determine the effect that use of Cell Collective has on student understanding, long-term retention, critical thinking, application, and overall mastery of material.

In addition to directly addressing the challenging problem of teaching students about complex, highly connected networks, there is an additional benefit; it provides these students with an opportunity to become interested in additional training in computational methods, something that is critical for the current and the next generation of biomedical researchers. Making the class accessible for students with a wide range of skills such as biology, computer science, mathematics, etc., creates an ample environment for learning from one another, resulting in cross-pollination across disciplines.

Furthermore, one of the major components of utilization of Cell Collective was blurring the line between learning and research. This is a non-trivial aspect of this teaching method and, indeed, it is in some ways the most exciting—for both the students and potentially the instructors. In the course of their learning, students have the opportunity to be constructing the very first model of the system they are studying or, if working on an existing model, they have the opportunity to add information from recent literature to significantly update an existing model. This means that students, while learning, are engaging in real research. In our application of this teaching method, we have had a number of student-created and/or student-initiated modeling projects that led to research findings, some of which were subsequently presented by the students at an external research conference [28], and even accepted for publication in a peer-reviewed journal [29].

A positive consequence of this is that it is possible for faculty to further their own research during the course of teaching the material. We have had several experiences of students who had no knowledge of what they might be interested in studying being assigned a project in the class that aligned with a research interest of our group. In several cases, the results were ultimately useful to the group, and in subsequent semesters new students were assigned to either re-create, significantly update, or provide fresh analysis of the model.

All students that made significant contributions to the models were included as authors on all publications using that information. This result is a true win-win situation; faculty responsible for teaching a course have the possibility of actually furthering their research, while students have the possibility to perform and be recognized for research participation. Real undergraduate research is not only a major goal for many universities, it is also very important for any undergraduate looking for entry into graduate programs.

## Supporting Information

S1 Text Integrating interactive computational modeling in biology curricula. (PDF)
