# Supplementary material for: Integrating Interactive Computational Modeling in Biology Curricula
Source: PLoS Comput Biol. 2015 Mar 19;11(3):e1004131. doi: 10.1371/journal.pcbi.1004131 (PMC4366374; doi:10.1371/journal.pcbi.1004131)
Supplement: S1 Text — (PDF) [file pcbi.1004131.s001.pdf]

Integrating interactive computational modeling in biology curricula: Supporting Text 1.

Section 1: Technology and Modeling Framework for Cell Collective

Cell Collective (www.thecellcollective.org) is a web-based platform for the creation and simulation of computational models of biological/biochemical processes [11,12]. The mathematical framework utilized in the platform is based on logical discrete networks first developed by S. Kauffman [15] and R. Thomas [16] In these networks, components (e.g., genes or proteins) assume discrete values representing their activity levels (e.g., gene expression). In Boolean networks individual nodes can represent any component of the system (e.g., genes in gene regulatory networks), while edges correspond to the biochemical/biological interactions between the components. Each component (represented as a circle in the illustration) of the network can assume either an active or inactive state. As the model evolves in time, the state of each model component is updated based on its Boolean function, which describes the regulatory mechanism of a given network component [17-19].

Modeling approaches range from ones using differential equations, able to capture high levels of detail, to more coarse-grained approaches such as Boolean networks. The rich information of kinetics contained in differential equations comes with the price of lijmited scalability, and dependency on many difficult-to-obtain biological (kinetic) parameters. In the classroom setting, these methodologies typically require strong knowledge of differential equations that many biology students lack and/or fear these concepts. On the other hand, Boolean networks are based on the logic of individual interactions, free of kinetic parameters, and relatively easy to construct, while providing a way to study the dynamics of complex non-linear systems. These attributes make this approach suitable for the modeling of large-scale systems and, as exemplified in this paper, for the application in biology courses due to the relatively low barrier of entry for students with limited background in mathematics.

Figure S1 illustrates an example of a hypothetical three-component network. The regulatory mechanism of each component is defined by a Boolean function, which can be represented with a “state look-up table”. The left side of each table contains all possible active/inactive state combinations for all regulators of a given component. For instance, the table for component Y has two columns for each of the regulators, X and Z, and four rows containing all four possible state combinations. On the other hand, the state look-up table for X has only one column (one regulator, Y) and two rows (because Y can be only active or inactive). The right column of the table depicts the regulatory mechanism of the component under the combinational conditions of the upstream regulators listed on the left side of each table. For example, from the state look-up table for component Y, it will be active when both or either of X and Z are active.

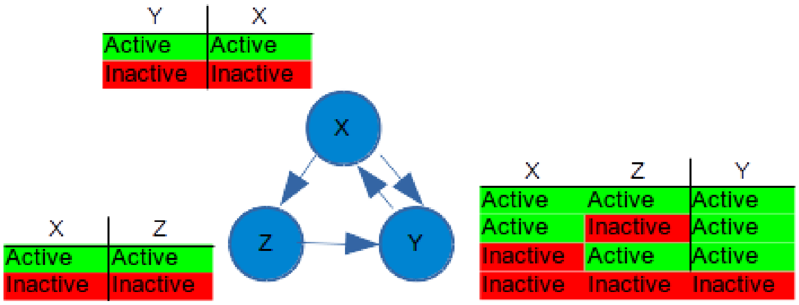

Figure S1: An example of a hypothetical three-component Boolean network

Section 2: Bacterial chemotaxis: A simple first dynamical system to investigate via computational modeling

Students are taught the basic principles of chemotaxis; specifically, that chemotaxis is a fundamental

function of single-celled organisms that allows them to move in space in response to various soluble environmental factors [1,2]. The mechanism by which this happens involves propulsion of the cells via the rotational movement of a flagellar tail that extends from its body. The rotation of the tail is achieved by molecular motors in the cell body by varying the direction of rotation (clockwise results in tumbling/turning, counter-clockwise results in straight-line movement). This allows them to sense and respond to concentration gradients of chemoeffectors such as food sources or toxins and adjust their movement (toward or away) appropriately. The motors that determine the rotational direction of the flagellum (and, thus, the direction of movement of the cell) are regulated by protein networks that serve as the interface between the chemoreceptors on the surface of the cell and the motors.

The protein signaling network responsible for controlling flagellar rotation consists of a relatively small number of proteins (Figure S2A&B), yet is complex enough to demonstrate nontrivial phenomena. This makes it an ideal system for students to learn how network dynamics are involved in biological phenomena. In studying chemotaxis, students learn not only the biochemistry governing the biochemical network regulating movement, but also how a negative feedback loop in such a network produces oscillations, which can result in *adaptability* of the bacterium to its environment. The property of adaptability allows the bacterium's signaling network to process and store information about the strength of the last signal received and "remember" the previous position in its environment (i.e., the amount of the chemoattractant), resulting in the bacterium's ability to sense whether it is moving along or against the chemoattractant gradient [3,4].

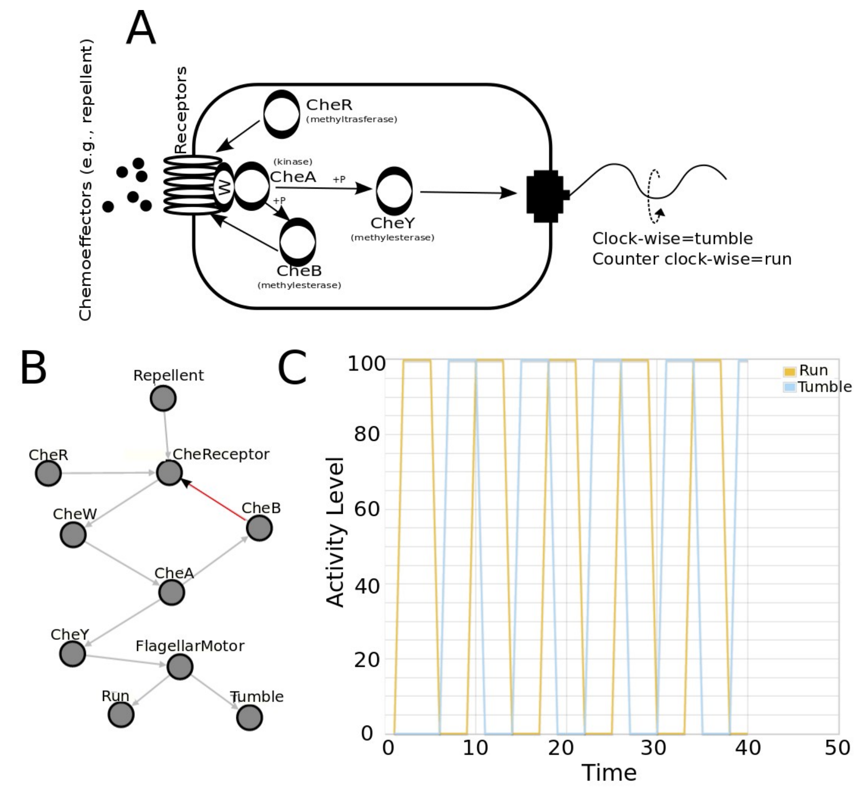

Figure S2. Bacterial chemotaxis and its model representation in Cell Collective. A) Bacterial chemotaxis illustration. In response to various levels of chemoattractors binding of chemoreceptors, the network increases or decreases the level of methylation of the motors, and that in turn controls the direction of the motor's rotation; high levels of chemoattractor binding results in a decreased motor methylation (inducing the bacterium's straight-line motion), while lower concentrations (or absence) of chemoattractants, results in higher levels of methylation of the receptor and reverses the rotation, resulting in tumbling/turning motion. B) Network representation of the biochemical interactions regulating the flagellar motor and the direction of its rotation. The red arrow represents a negative regulation of the chemokine receptor by CheB, forming a negative feedback look (CheReceptor → CheW → CheA → CheB -| CheReceptor. C) Screen-shot of a real-time simulation of the chemotaxis network model in Cell Collective, representing the biochemical network in panel B illustrating the oscillatory chemotaxis dynamic responsible for the adaptive abilities of bacteria.

Section 3: Learning by modeling – Reading the literature and the challenge of creating a functional model

Consider the biochemical regulatory mechanism of a hypothetical molecular component (e.g., protein) *X*, whereby *X* has two regulators, one positive regulator (activator), *A,* and an inhibitor, *B* (Figure S3). The table

in Figure S3 represents the activity combinations of *A* and *B* under which *X* will be active. In other words, this state look-up table represents the regulatory mechanism of *X*.

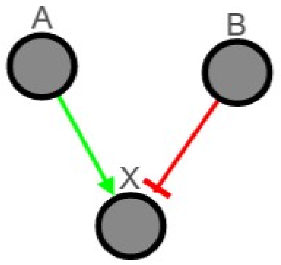

A	B	X
Inactive	Inactive	Inactive
Inactive	Active	Inactive
Active	Inactive	Active
Active	Active	?

Figure S3: Hypothetical example of a biochemical regulatory mechanism and its network representation. See text for detailed description.

From this simple example, the first three activity combinations in the table are clear from the provided description of the regulation of *X* (similar data are also readily available in published papers for many real biological components). Specifically, when both regulators are inactive, *X* will be also inactive; when only the activator *A* is active, *X* will be active, and when only the inhibitor *B* is active, *X* will be inactive. However, what will the activity of *X* be when *both A* and *B* are active? This information is necessary in order to complete and simulate a model, but the aforementioned regulatory mechanism of *X* did not explicitly provide this knowledge. Similar questions are common, and answering them requires more detailed research of the regulatory mechanism of the component by the student.

For example, it could be the case that *A* is a kinase that activates *X* upon its phosphorylation, and *B* is a phosphatase that inactivates it via dephosphorylation of the same site. In such a case, the inhibitor would be dominant over the activator, and *X* would be inactive when both *A* and *B* are active. In contrast, if the biochemical effects of *A* and *B* on *X* were the result of post-translational modification on two different sites, *X* could be considered active when both *A* and *B* are active. Because such deep knowledge about each component of the network is necessary to create a model, students learn the detailed regulatory system of the researched biological/biochemical process. If all detail to model a complete regulatory mechanism is not available in published papers, students can play with the model to figure out the best possible solution, given the available literature.

In addition, students learn about the dynamics of the modeled processes during the model-testing phase. This is generally done by testing the model for the ability to reproduce previously published, laboratory-based, input-output phenomena. For example, when modeling a receptor tyrosine kinase (RTK), integrin, and G-protein coupled receptor (GPCR) pathways [5], we used RTK activation of Erk with a dependence on integrin signaling as a general phenomenon that the system should reproduce, as well as trans-activation of RTKs by GPCR signaling. As such, students augment their learning about the details of the individual components of the process with a better understanding of the overall dynamics that arise from the individual components working together. Following this process, students become extremely knowledgeable of the literature and functional interactions of the network they are studying. Not only do they know what is in the literature, their empirical work can often lead to insights into the system that can actually become the basis of new research and opportunities for publications (e.g., [29]) and/or research presentations at national undergraduate research conferences (in one case a student was honored with a first place award in the "Physiology and Immunology" topic for her research in signal transduction CD4+ T cells by using the discussed modeling approach) [6].

Section 4: Evaluation

In Silico Biology. In the first half of the course, students are evaluated based on assignments and an exam that test students' understanding of the modeling approach, as well as their ability to describe (and model) a complex regulatory mechanism of a given enzyme. The second half of the course centers around a project (#3 above) that is evaluated based on a submitted project topic abstract, two to three presentations, and a final paper.

The model building process involves first the identification of local biochemical/biological interactions (from published literature) that comprise a regulatory mechanism of a model component. Similar to models created in the context biomedical research, the model needs to be validated by simulating global, complex, behaviors as observed in the laboratory. One cannot expect that *all* phenomena will be replicated by the

model due to the fact that the model will be missing other components and/or may contain certain components that are based on incorrect/missing laboratory data, or because the student misinterpreted the data. The reproducibility of certain phenomena by the model provides students with indications that the model is on the right track. The non-working behaviors, in turn, serve as indicators to further review the model's local interactions (and/or as new hypotheses for further laboratory research in the case of research application). Part of the assessment is the ability of the student to show how well the model works, as well as to identify phenomena that do not work as expected and hypothesize as to why these phenomena could not be replicated by the model (as opposed to the need to review every individual interaction and citation used to synthesize the model). This becomes evident through student presentations, as well as their final term paper. It is also important to note that in addition to learning about the given biological process, students are able to experience the exact method of how computational models are synthesized in a real research setting.

As such, the model synthesized and studied as part of the project is evaluated based on its complexity and the student's ability to formulate how the model is able to reproduce previously reported (holistic) phenomena (validation), as well as the model's limitations, and potential reasons for the shortcomings (e.g., conflicting laboratory data, pathways missing in the model, etc.)

Cancer Biology and Immunology courses. Students that used the Cell Collective were compared to course-matched controls (i.e. those that did not use Cell Collective based on propensity score matching, PSM). The answers to the same questions assessing student learning objectives 1 and 2 (see main text) for microbiology were scored by 2 independent researchers as compared with the control population. Students that used Cell Collective showed an increased understanding (Figure S4). While this result currently lacks statistical significance, this finding was substantiated by the fact that students that used Cell Collective also asked more questions of faculty (C.E.C. & T.H.) regarding the biological life cycles illustrated in the microbiology course. We plan continued follow-up studies in order to build a better picture based on a larger sample size.

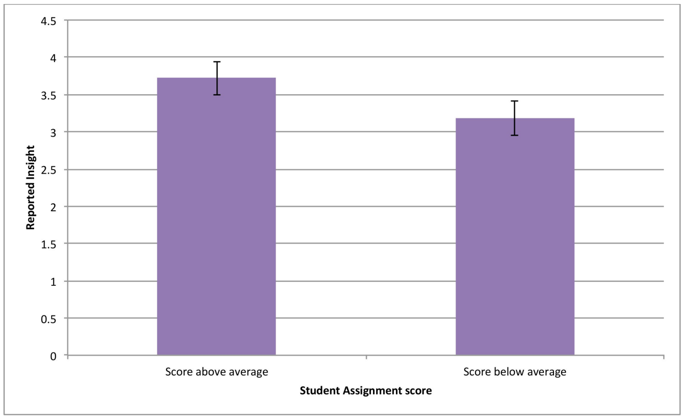

Figure S4: Students who used Cell Collective scored higher than those who did not use the modeling approach.

Section 5: Student Feedback

Students from the *In Silico Biology* course as well as immunology, microbiology, and cancer biology classes were surveyed (Tables S1 and S2) to obtain their feedback on the use of computational modeling and Cell Collective as a teaching and learning method[1]. Surveys were completed by students at the end of each course on a voluntary basis (and rewarded by few extra credit points).

Table S1. Survey questions used in the cancer biology course.

On a 0 (none) - 5 (expert) scale, how would you rate your expertise in network modeling before AND after the course:						
Before:	0	1	2	3	4	5
After:	0	1	2	3	4	5

The hands-on, computer modeling-based assignments were beneficial to my understanding of the presented material.<br><br>Strongly Agree Agree Neutral Disagree Strongly Disagree
I found the computer modeling-based assignments to be interesting.<br><br>Strongly Agree Agree Neutral Disagree Strongly Disagree
The modeling-based assignments provided new insights into the biological process(es) of cancer biology.<br><br>Strongly Agree Agree Neutral Disagree Strongly Disagree
I would enjoy more modeling-based assignments in other classes.<br><br>Strongly Agree Agree Neutral Disagree Strongly Disagree
As a result of the modeling-based approach, I plan to try to use the software in other classes or my research whether or not is required.<br><br>Strongly Agree Agree Neutral Disagree Strongly Disagree
Would say that using the Cell Collective software was:<br><br>Very Easy Easy Average Difficult Very Difficult

Table S2. Sample survey questions used in the In Silico Biology course.

Describe in 1-3 sentences what you found most interesting in this class (and why).
Describe in 1-3 sentences what you found least interesting in this class (and why).
Describe in 1-3 sentences whether, overall, this class gave you new insights into *biological systems*. In other words, was this just another class to you, or was the hands-on approach that actually helped you to better understand biological systems? If it did help you to understand biology better, provide additional 1-3 sentences to explain why/how, but be general enough that I will not be able to identify you through your response.
Describe in 1-3 sentences whether, overall, this class gave you new insights into *modeling and simulation* concepts. In other words, do you feel like you now have an understanding of new ideas as to how to abstract biological knowledge into a model (i.e., virtual cell) that you did not have before?
In one sentence, indicate whether you would recommend other biology majors to take this class. In 1-2 sentences explain why.

Feedback collected from *In Silico Biology* students (sample of 31) has been consistent and encouraging. When asked what part of the course was the most interesting, 40% students indicated the ability to model the discussed biological systems. The majority of students indicated their appreciation to be able to view and study biological processes from a systems view, ability to visualize their dynamics, and the ability to tinker with the models. Fifteen percent of students most enjoyed the concepts of logic and Boolean networks. As some would expect, several students (15%) commented on the tedium of researching literature in order to construct a model; however, these students also acknowledged the necessity for this step to construct a model, and to better understand the biology behind it.

The initial integration of modeling modules into general as well as specialized biology courses has been applied in a graduate immunology (on-line) course, an undergraduate (on-line) cancer biology course, and traditional (in-class) microbiology course. The salient findings from student surveys and exit interviews[1] from

1 In concordance with the Institutional Review Board at the University of Nebraska, all completed surveys were analyzed blindly (IRB-EX-548-12; C.E.C., applicable to the immunology and microbiology courses). Additionally, student codes were used rather than name or student identification numbers as identifiers. Both undergraduates (*n*=24) and graduate students (*n*=18) in the immunology and microbiology courses were surveyed for their feedback on the Cell Collective regarding the ease of use, supplement to learning, and translation/application.

immunology and microbiology indicate that the majority of students (~80%) found the ability to view the dynamics of the taught biological concept useful, and improved learning as a supplement or *in lieu* of a traditional textbook-/lecture-based approach. Some students found the models as a good way to reiterate and revisit concepts presented in the textbook or during lecture, including assistance in test preparation. Other students also reported using the software voluntarily for studying topics that did not have a modeling component. Over half of the surveyed students (53%) found the software initially intimidating, but easy to use once a few practice simulations were done. We believe that the initial intimidation was largely due to the fact that the students were being introduced to a dramatically different way of learning biology through computer simulation which can also generate apprehension.

To minimize any learning curves associated with the modeling concepts and the software, we have developed a series of simple, introductory modeling exercises designed to first introduce students to the concepts. These exercises have been developed for cancer biology topics (available as a workbook in [21]) and were most recently implemented in a cancer biology course. Students were asked to fill out a survey at the end of this course and, in fact, 71% of the surveyed students (n=7) liked the new exercise-based format, and some (43%) also suggested videos as a format for the introduction of modeling exercises. All surveyed students who used Cell Collective in the cancer biology course self-reported that their expertise in network modeling improved (Figure S5), and the hands-on, computer-modeling assignments were beneficial to their understanding of the presented material and providing new insights into the biological processes of cancer biology (Figures S6 and S7). Furthermore, 86% of these students found the modeling-based assignments interesting (Figure S8), and 43% of the students would enjoy more modeling-based assignments in other courses (Figure S9). In addition, 57% of the students indicated that they will apply this modeling approach and software in other courses or their research projects regardless of whether it is required (Figure S10). Finally, only one student found Cell Collective difficult to use (Figure S11). All students also provided invaluable feedback on how to improve the software as well as the presentation and implementation of the model-based learning exercises that we plan to integrate in the next versions of our courses.

Furthermore, for the Cancer Biology and Immunology courses, students that used the Cell Collective were compared to course-matched controls (i.e. those that did not use the Cell Collective based on propensity score matching, PSM). The answers to the same questions assessing student learning objectives 1&2 for microbiology were scored by two independent researchers as compared with the control population. Students that used the Cell Collective showed an increased understanding. This finding was substantiated by the fact that students that used the Cell Collective also asked more questions of faculty (C.E.C. & T.H.) regarding the biological life cycles illustrated in the microbiology course.

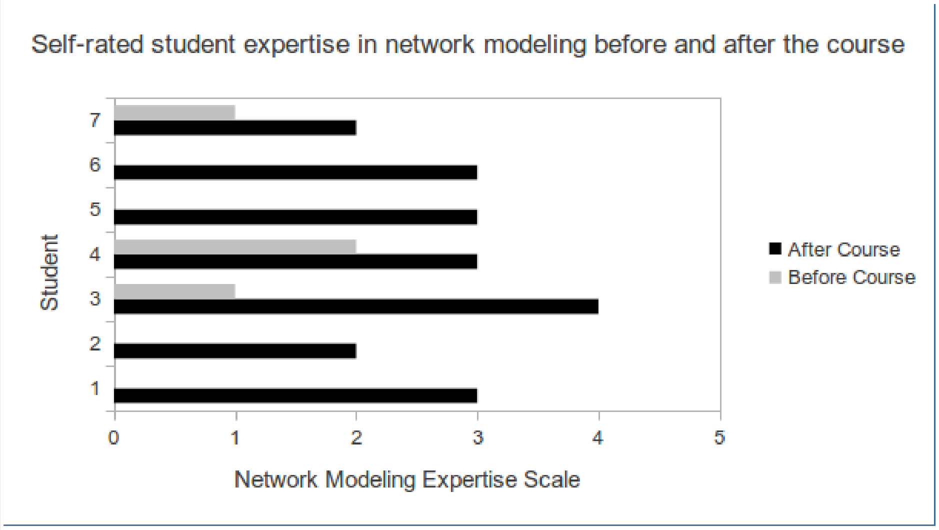

Figure S5: Self-rated student expertise in network modeling before and after the course. N=7; Students were offered extra credit to complete the optional surveys.

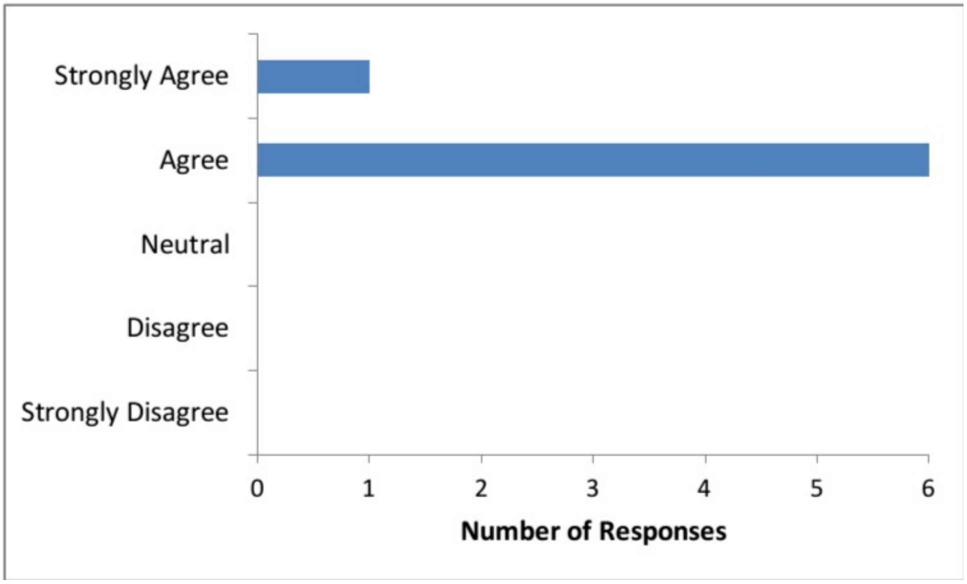

Figure S6. Student responses to whether they found the hands-on, computer modeling-based assignments beneficial to their understanding of the presented material.

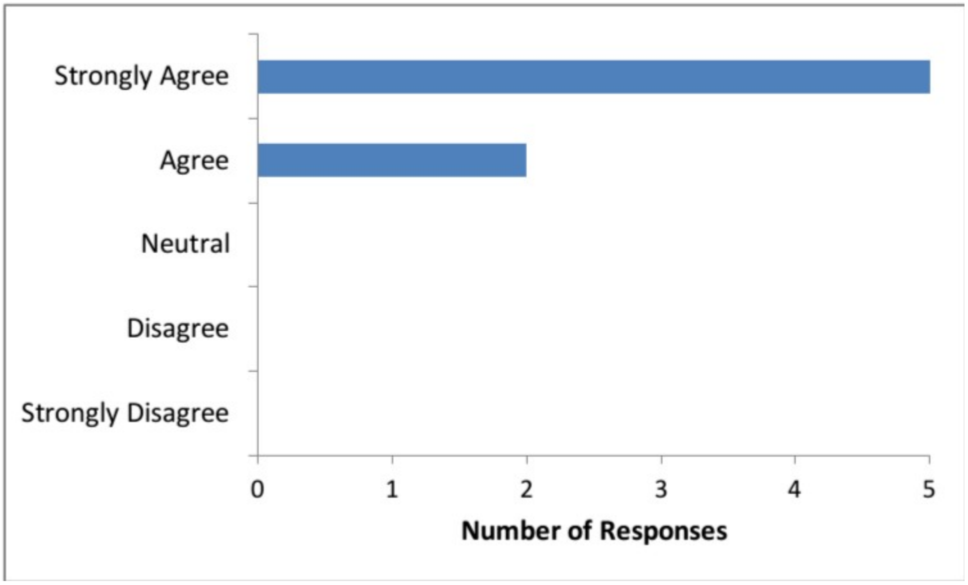

Figure S7. Student responses to whether the modeling-based assignments provided new insights into the biological process(es) of cancer biology.

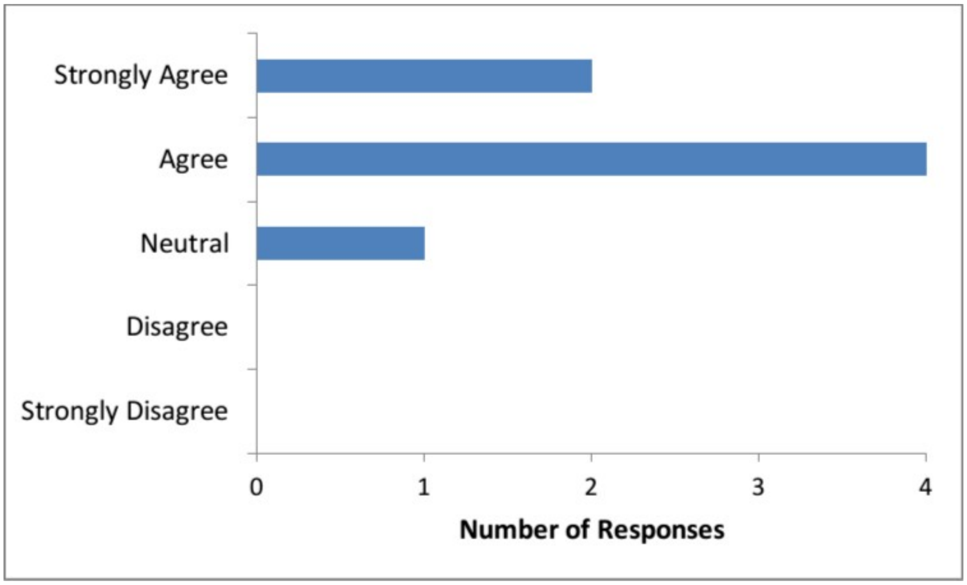

Figure S8. Student responses to whether they found the computer modeling-based assignments to be interesting.

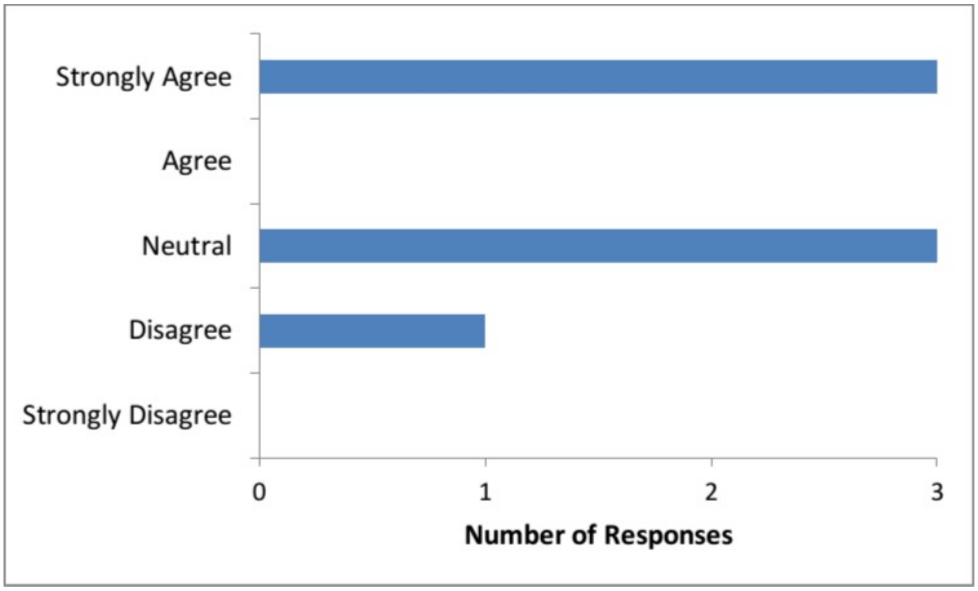

Figure S9. Student responses to whether they would enjoy more modeling-based assignments in other courses.

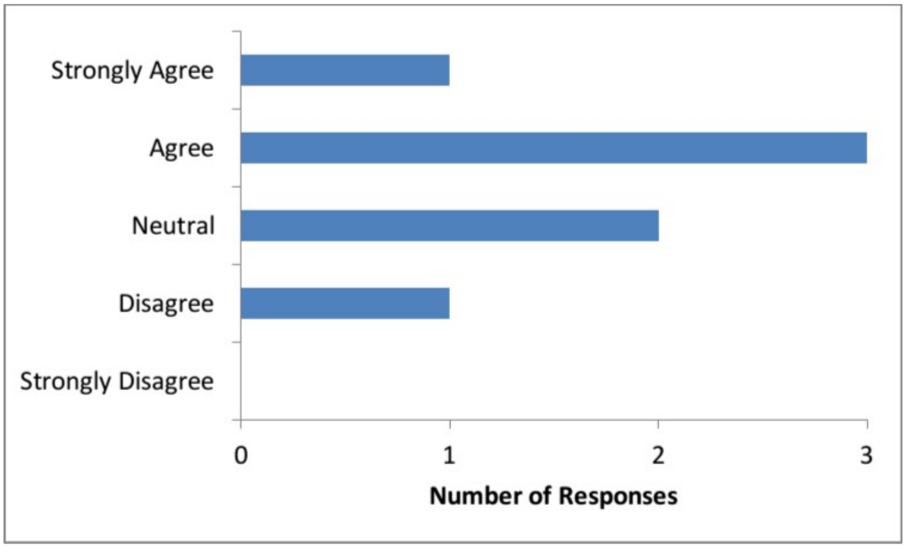

Figure S10. Student responses to whether they would try to use the software in other classes or research projects whether or not it was required.

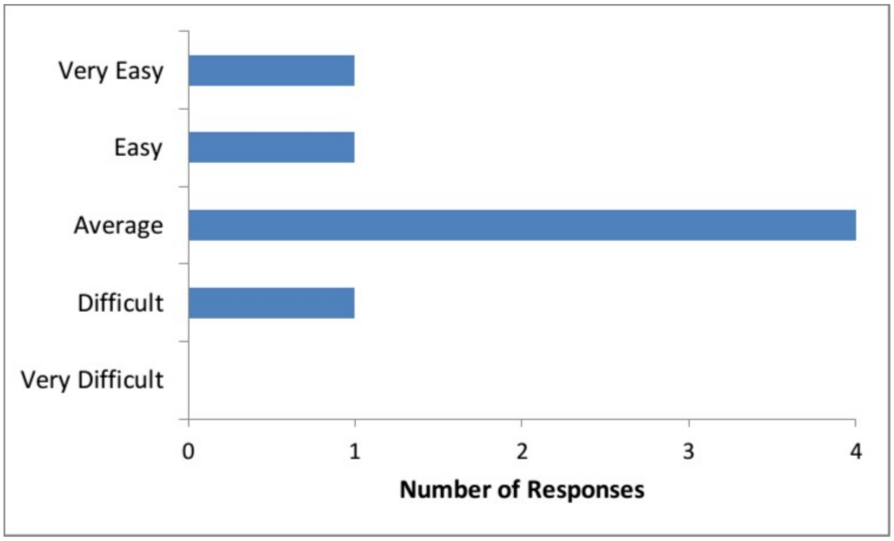

Figure S11. Student ranking of the ease-of-use of Cell Collective.

References Cited

1. Blair DF. How bacteria sense and swim. Annu Rev Microbiol. Annual Reviews 4139 El Camino Way, P.O. Box 10139, Palo Alto, CA 94303-0139, USA; 1995;49: 489–522. doi:10.1146/annurev.mi.49.100195.002421

2. Rao C V, Kirby JR, Arkin AP. Design and diversity in bacterial chemotaxis: a comparative study in Escherichia coli and Bacillus subtilis. PLoS Biol. 2004;2: E49. doi:10.1371/journal.pbio.0020049

3. Bourret RB, Stock AM. Molecular information processing: lessons from bacterial chemotaxis. J Biol Chem. 2002;277: 9625–8. doi:10.1074/jbc.R100066200

4. Stock JB, Levit MN, Wolanin PM. Information processing in bacterial chemotaxis. Sci STKE. 2002;2002: pe25. doi:10.1126/stke.2002.132.pe25

5. Helikar T, Konvalina J, Heidel J, Rogers JA. Emergent decision-making in biological signal transduction networks. Proc Natl Acad Sci U S A. Department of Pathology and Microbiology, University of Nebraska Medical Center, 983135 Nebraska Medical Center, Omaha, NE 68198, USA.; 2008;105: 1913–1918.

6. Sigma Xi, The Scientific Research Society: Student Research Conference [Internet]. 2013 [cited 21 Feb 2014]. Available: http://www.sigmaxi.org/meetings/src/2013src.shtml
